# The commercial determinants of health in Ireland: fueling an industrial epidemic at home and abroad

**DOI:** 10.3399/BJGPO.2024.0029

**Published:** 2024-05-15

**Authors:** Mélissa Mialon, James Larkin, Clare Patton, Mimi Tatlow-Golden, Kathryn Reilly, Paula Leonard, Malvina Walsh, Norah Campbell

**Affiliations:** 1 Trinity Business School, Trinity College Dublin, Dublin, Ireland; 2 Univ Rennes, EHESP, CNRS, Inserm, Arènes—UMR 6051, RSMS (Recherche sur les Services et Management en Santé) - U 1309, Rennes, France; 3 Department of General Practice, RCSI University of Medicine and Health Sciences, Dublin, Ireland; 4 School of Law, University of Leeds, Leeds, UK; 5 Faculty of Wellbeing, Education and Language Studies, The Open University, Milton Keynes, UK; 6 Irish Heart Foundation, Dublin, Ireland; 7 Alcohol Forum Ireland, Letterkenny, Ireland; 8 Citizen researcher, West Wicklow, Ireland

**Keywords:** Commercial determinants of health, public policy, public health, general practitioners, primary healthcare, marketing, corporate political activity, corporate social responsibility

More than half of deaths in Ireland are caused by four harmful commodities: tobacco, alcohol, fossil fuels, and unhealthy foods (Table 1). Commercial actors, through mechanisms that make these commodities artificially cheap, hyper-convenient, and seductively attractive, are a key driver for ill-health. The cumulative effects of these commercial vectors have been described in a landmark Series on the Commercial Determinants of Health (CDoH) by the eminent medical journal *The Lancet* in 2023.^1^ Here, we reflect on the findings and recommendations from the Series in the context of Ireland.

**Table 1.** Burden of annual deaths attributable to commercial products in Ireland. Our table was adapted from Gilmore *et al*. Lancet Series on Commercial Determinants of Health 2023.^10^ We used data for Ireland from the Global Burden of Disease Collaborative Network - Global Burden of Disease Study 2019 (GBD 2019) Results, Institute for Health Metrics and Evaluation (IHME). Data are available from https://vizhub.healthdata.org/gbd-compare/#. Results are underestimated, as explained in the Lancet Series

*Risks factors for deaths in Ireland*	*Number of annual deaths*
**Air pollution**	
Ambient ozone pollution	15.72
Ambient particulate matter pollution	535.45
**Alcohol**	1,543.08
**Dietary-related risks**	
Diet high in red meat	1,001.96
Diet high in processed meat	373.74
Diet high in SSB	169.50
Diet high in sodium	336.36
Sub-optimal breastfeeding	0.65
**Tobacco**	6,458.64
***All deaths***	18,655.16
***Deaths attributable to those commercial products in Ireland***	10,435.10 (56%)

In Ireland, alcohol consumption is responsible for three deaths per day,^2^ yet the alcohol industry lobbies government ministers, senior officials and Oireachtas members against the introduction of regulation on alcohol.^3^ The alcohol industry met 361 times with the Irish government in 2018 alone, the year the Dáil debated the Public Health Alcohol Act.^4^ The food industry, and its representative trade bodies, have access to officials in the Department of Health, exercising influence on issues such as taxation, marketing, and food labelling.^5^ Despite its conflicting interest with those of public health, the gambling industry, through the Gambling Awareness Trust (GAT),^6^ is fast establishing itself as one of the primary sources of funding for Ireland’s response to gambling harms. The pharmaceutical and medical device industries shape integral aspects of our health system to align with commercial interests, including medical education, medical procurement, research and development, open science practices, the patent system, and drug pricing.^7^ In addition, the private healthcare sector in Ireland has used its power to undermine efforts to provide universal access to healthcare.^8^

Ireland, like other countries across the globe, as discussed in the Lancet Series, faces an industrial epidemic, where commercial actors are responsible for the exposure of the Irish population to harmful commodities and commercial practices.^9^ The Lancet Series made it clear that the industrial epidemic is the face of a political economic system which benefits commercial interests at the expense of the human right to health.^10^ Towards the end of the last century, the Irish government pursued policies of deregulation and low corporate taxation.^11^ Though this has increased economic activity in the country, it has had concomitant negative externalities. Ireland’s low corporation tax deprives many countries (including low and middle income countries) of revenue that could be used to strengthen health and education systems.^12^

In that context, and given the harms to population health of the CDoH and the recommendations made in the Lancet Series, we call on health professionals and experts in Ireland to take action by:

- getting informed about the CDoH;
- engaging in training and capacity building on the topic;
- avoiding partaking in education sponsored by commercial actors;
- working in coalition to address the CDoH;
- lobbying for the introduction of mechanisms to protect public health policy from undue corporate influence; for example, lobbying organisations that you are a member or employee of to introduce stringent conflict of interest policies;
- avoiding payment for your work from commercial actors, particularly those with an interest conflicting with that of public health.

## Data

There is no data or software associated with this article.
